# High Yield Production and Refolding of the Double-Knot Toxin, an Activator of TRPV1 Channels

**DOI:** 10.1371/journal.pone.0051516

**Published:** 2012-12-11

**Authors:** Chanhyung Bae, Jeet Kalia, Inhye Song, JeongHeon Yu, Ha Hyung Kim, Kenton J. Swartz, Jae Il Kim

**Affiliations:** 1 School of Life Science, Gwangju Institute of Science and Technology (GIST), Gwangju, Republic of Korea; 2 Molecular Physiology and Biophysics Section, Porter Neuroscience Research Center, National Institute of Neurological Disorders and Stroke, National Institutes of Health, Bethesda, Maryland, United States of America; 3 College of Pharmacy, Chung-Ang University, Seoul, Republic of Korea; Sackler Medical School, Tel Aviv University, Israel

## Abstract

A unique peptide toxin, named double-knot toxin (DkTx), was recently purified from the venom of the tarantula *Ornithoctonus huwena* and was found to stably activate TRPV1 channels by targeting the outer pore domain. DkTx has been shown to consist of two inhibitory cysteine-knot (ICK) motifs, referred to as K1 and K2, each containing six cysteine residues. Beyond this initial characterization, however, the structural and functional details about DkTx remains elusive in large part due to the lack of a high yielding methodology for the synthesis and folding of this cysteine-rich peptide. Here, we overcome this obstacle by generating pure DkTx in quantities sufficient for structural and functional analyses. Our methodology entails expression of DkTx in *E. coli* followed by oxidative folding of the isolated linear peptide. Upon screening of various oxidative conditions for optimizing the folding yield of the toxin, we observed that detergents were required for efficient folding of the linear peptide. Our synthetic DkTx co-eluted with the native toxin on HPLC, and irreversibly activated TRPV1 in a manner identical to native DkTx. Interestingly, we find that DkTx has two interconvertible conformations present in a 1∶6 ratio at equilibrium. Kinetic analysis of DkTx folding suggests that the K1 and K2 domains influence each other during the folding process. Moreover, the CD spectra of the toxins shows that the secondary structures of K1 and K2 remains intact even after separating the two knots. These findings provide a starting point for detailed studies on the structural and functional characterization of DkTx and utilization of this toxin as a tool to explore the elusive mechanisms underlying the polymodal gating of TRPV1.

## Introduction

Spider venom is a cocktail containing a variety of compounds, including small molecules, peptides and proteins [1]–[6]. These components play an important role in prey capture and defense against predators and competitors by binding to membrane proteins such as ion channels and receptors within the nervous system [7], [8]. These peptides disrupt proper ion channel function to induce paralysis through direct blockade or induced release of neurotransmitters [9]–[11]. Several such peptide toxins such as omega-agatoxin IVA [12], [13], VsTx1 [14], [15] and omega-atracotoxin-HV1 [16], have been purified and used to target various ion channels (e.g., calcium, sodium or potassium channels). Recently, a peptide toxin named double-knot toxin (DkTx) was purified from the venom of the tarantula *Ornithoctonus huwena* [17]. DkTx activates the TRPV1 channel by binding directly to the outer pore domain with relatively high affinity and slow dissociation kinetics. Composed of 75 amino acids, DkTx shows a novel sequence feature: two inhibitory cysteine-knot (ICK) motifs named knot1 (K1) and knot2 (K2) domains. DkTx the first peptide toxin known to contain two ICK motifs within one molecule and highlights the genetic diversity of toxins originating from terrestrial and marine predators.

DkTx is only the third type of peptide toxin that has been reported to modulate TRPV1 function. Vanillotoxins [18], were the first such toxins discovered, were shown to activate TRPV1 channels. Thereafter, APHC1 [19] was shown to inhibit capsaicin-induced currents in TRPV1-expressing cells. The structural and functional information about these TRPV1-targeting peptides is still scarce, in part because synthesis of these cysteine-rich peptides is limited due to their very low efficiency of folding. Thus, a better understanding of these peptides will require improvement of the synthetic conditions. As a first step toward studying the structure-activity relationships of DkTx, we sought to develop a high-yield expression system for the toxin, optimize and characterize *in vitro* folding conditions, and test the activity of toxin constructs against the TRPV1 channel using electrophysiological approaches.

## Materials and Methods

### Expression of DkTx Using Different Fusion Proteins

We used overlapping PCR to synthesize an artificial DkTx gene [20], in which the DNA codons were optimized for efficient expression in *E. coli*. This synthetic gene was amplified by PCR and cloned into pET28a, pET31b or pET32a vector. The primer pair 5′- aaacgcCTCGAGAACGGCGATTGCGCG-3′ and 5′-aaacgcCTCGAGTTAGCGATATTTTTCGCAATACG-3′ were used for cloning into pET31b vector; and 5′-aaacgcGGATCCAACGGCGATTGCGCG-3′ and 5′- aaacgcCTCGAGTTAGCGATATTTTTCGCAATACG-3′ were used for cloning into pET28 and pET32a vectors. The underlined bases contain restriction sites, and bases in lower case are extra bases for efficient cleavage by the restriction enzyme.

**Table 1:** Oxidative folding yield of DkTx under different conditions^a^.

Condition	Buffer condition^b^	Additive	Folding yield (%)^c^	Temperature (°C)
1	A	–	2.27	4
2	A	1 M GdnHCl	3.65	4
3	A	10% glycerol	2.00	4
4	A	20% EtOH	1.30	4
5	A	10% MeCN	5.27	4
6	A	20% MeCN	7.22	4
7	A	40% MeCN	7.90	4
8	B	–	2.27	4
9	B	1 M GdnHCl	2.86	4
10	B	10% glycerol	1.87	4
11	B	20% EtOH	1.20	4
12	A	0.5% Tween20	11.17	4
13	A	0.5% NP40	18.83	4
14	A	0.5% Triton X-100	24.08	4
15	A	0.5% DM	24.21	4
16	A	0.5% DDM	27.81	4
17	A	0.5% Fos-choline	29.85	4
18	A	0.5% Triton X-100	2.31	37

a Each folding condition contains 1 mM EDTA, 2.5 mM GSH and 0.25 mM GSSH. The concentration of DkTx in the folding solution was 12.5 µM.

b A: 0.4 M Tris-HCl (pH 8.0), B: 1 M NH_4_OAc (pH 8.0).

c Folding yield was calculated by comparing the areas of the HPLC peaks for the linear and folded toxin (peak area of correctly folded toxin × 100/peak area of linear toxin).

### Purification of Native DkTx

Native DkTx was purified from *Ornithoctonus huwena* venom (Spider Pharm) using an analytical C-18 reverse-phase high-performance liquid chromatography (RP-HPLC) column (HiChrom, ULT 5ODS) and a linear gradient from 5% CH_3_CN in water with 0.1% TFA to 65% CH_3_CN in water with 0.1% TFA over 30 min. Using this procedure, DkTx can be purified in a single step because the toxin elutes in region of the chromatogram that is relatively free of contaminating peptides.

**Table 2:** Folding properties of DkTx and its analogues.

Analogue	*k* (h^−1^)	Steady-state (%)¶	Activity, Kd (µM)
DkTx	0.046	50.20	0.28
K1	0.092	65.10	*
K2	0.103	30.16	1.57

¶ Steady-state (%) indicates the steady-state accumulation of the correctly folded form.

* The Kd for K1 could not be obtained because it was not possible to obtain saturable activation of TRPV1 by K1 (see Fig. 6).

### Production of Recombinant DkTx

*E. coli* BL21 (DE3) cells transformed with one of the aforementioned expression vectors were cultured at 37°C in LB media with suitable antibiotics. Once the OD_600_ reached 0.5–0.8, expression was induced by adding 0.5 mM isopropyl-1-thio-β-D-galactopyranoside. The cells were then incubated for an additional 4 h, harvested, resuspended in 50 mM Tris-Cl (pH 8.0), and ultrasonicated. The resultant cell lysate was centrifuged at 12,000 rpm for 1 h, after which the protein pellet was dissolved in 6 M GdnHCl to a concentration of 1 mg/ml. To cleave the fusion protein, hydroxylamine was added to a final concentration of 2 M, and the pH of the solution was adjusted to 9.0 [21]. After incubating the solution for 6 h at 45°C, the reaction was stopped by adjusting the pH to 3.5. One hour before stopping the reaction, dithiothreitol (DTT) was added to a final concentration of 300 mM to reduce the disulfide bonds of the misfolded protein. The reduced peptides were loaded onto a C18 column, and nonadsorbed components were washed away with water. The proteins remaining in the column were eluted using 70% CH_3_CN containing 0.1% trifluoroacetic acid (TFA), after which the eluate was lyophilized. Linear DkTx was then purified using semi-preparative RP-HPLC C-18 column (Shim-pak, Shimadzu).

### Peptide Synthesis

The single knots peptides (K1 and K2) were independently synthesized using solid-phase peptide-synthesis methods with Fmoc-chemistry. The linear peptides were cleaved from the resin by treatment of reagent K (TFA/water/ethanedithiol/phenol/thioanisole; 90∶5∶2.5∶7.5∶5) for 5 h, precipitated with ice-cold diethyl ether and washed three times to remove scavengers. The deprotected peptide was extracted with 50% CH_3_CN containing 0.1% TFA, the integrity of the peptide was validated by matrix assisted laser desorption/ionization time-of-flight mass spectrometry (MALDI-TOF MASS), and the peptides were purified by semi-preparative RP-HPLC.

### Oxidative Folding and Screening for Recombinant DkTx

The recombinant linear DkTx was dissolved in 50% CH_3_CN containing 0.1% TFA to a concentration of 1 mg/ml. The folding reaction was initiated by adding linear DkTx solution to 1.0 M ammonium acetate buffer (pH 8.0) or 0.4 M Tris-HCl buffer (pH 8.0), each containing 1 mM EDTA and reduced and oxidized glutathione (2.5 and 0.25 mM, respectively). The final concentration of linear DkTx in each folding solution was 12.5 µM. To test the effect of detergents and organic solvents on folding, these additives were introduced into the refolding mixture (see Table 1 for details) The folding reaction was allowed to run for 5 days, and after reaching equilibrium, the reaction was monitored using analytical RP-HPLC. The folding yield was calculated by comparing the areas of the HPLC peaks for the linear and folded toxins.

### Optimal Folding Conditions for DkTx, K 1, and K 2

Linear DkTx, K1 and K2 were separately dissolved in 50% CH_3_CN containing 0.1% TFA to a concentration of 1 mg/ml, after which the folding reaction was initiated by adding the linear toxin to the folding solution. The final composition of the folding solution was 0.4 M Tris-HCl (pH 8.0), 0.5% Triton X-100, 1 mM EDTA, 2.5 mM GSH, 0.25 mM GSSG and 0.004 mM toxin. The resulting mixture was incubated at 4°C until equilibrium was reached. At selected time points, a small amount of the solution was withdrawn and acidified by adding 1/10 volume of acetic acid to monitor the folding reaction. Correctly folded DkTx was purified by cation exchange chromatography by using the SP-5PW column (purchased from Tosoh Biosciences LLC) and semi-preparative RP-HPLC.

**Figure 1:**
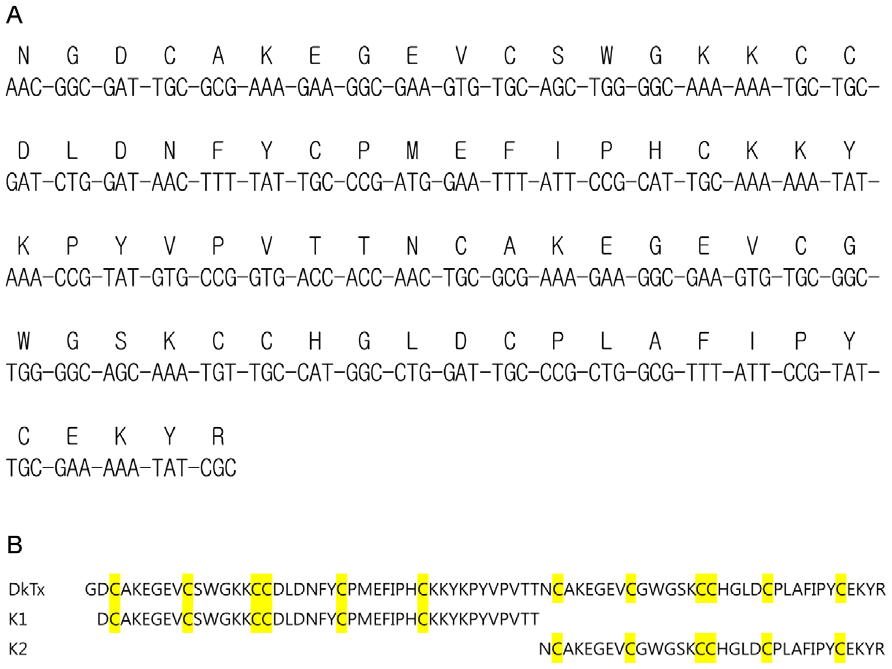
DNA construct encoding DkTx. (A) Design of a synthetic DkTx gene. (B) Sequence of the toxins used in the experiments. Note that DkTx has an N-terminal Gly residue because of the hydroxyl amine cleavage of Asn-Gly sequence added to the N-terminus.

**Figure 2:**
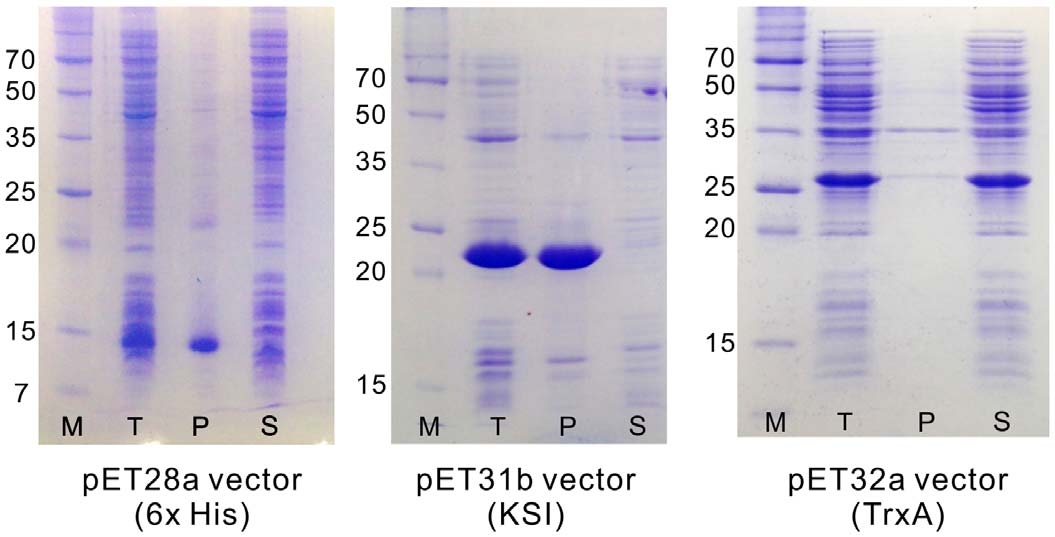
Expression of DkTx in *E. coli*. SDS-PAGE gels (15%) were stained with Coomassie Blue. Lane M, molecular weight markers (kDa); lane T, total cell lysate; lane P, pellet fraction; lane S, soluble fraction.

### Oocyte Preparation and Two-Electrode Voltage Clamp Recording

Oocytes were surgically removed from anesthetized Xenopus laevis frogs in accordance with protocol 1253-12 approved by the National Institute of Neurological Disorders and Stroke Animal Care and Use Committee. Oocytes were gently shaken for 60 min in a solution of 82.5 mM NaCl, 2.5 mM KCl, 1 mM MgCl_2_, 5 mM HEPES and 2 mg/mL collagenase. A rat TRPV1 construct (generously provided by D. Julius, UCSF) was cloned into the pGEM-HE vector, and used to generate cRNA. The cRNA was then injected into oocytes, which were then incubated for 1–3 days at 17°C in ND-96 solution (96 mM NaCl, 2 mM KCl, 5 mM HEPES, 1 mM MgCl_2_ and 50 µg/mL gentamycin, titrated to pH 7.6 with NaOH). TRPV1 activity was recorded under voltage clamp using a two-electrode voltage clamp (OC-725C; Warner Instruments) in a 150-µL recording chamber. The recorded data were filtered at 1 kHz and digitized at 5 kHz using a digidata analog/digital converter and pClamp software (Molecular Devices). Microelectrode resistances were 0.1–1 MΩ when filled with 3 M KCl. The external recording solution contained 115 mM NaCl, 2.5 mM KCl, 1.5 mM MgCl_2_ and 10 mM HEPES, titrated to pH 7.4 with NaOH. All experiments were performed at room temperature (∼22°C).

**Figure 3:**
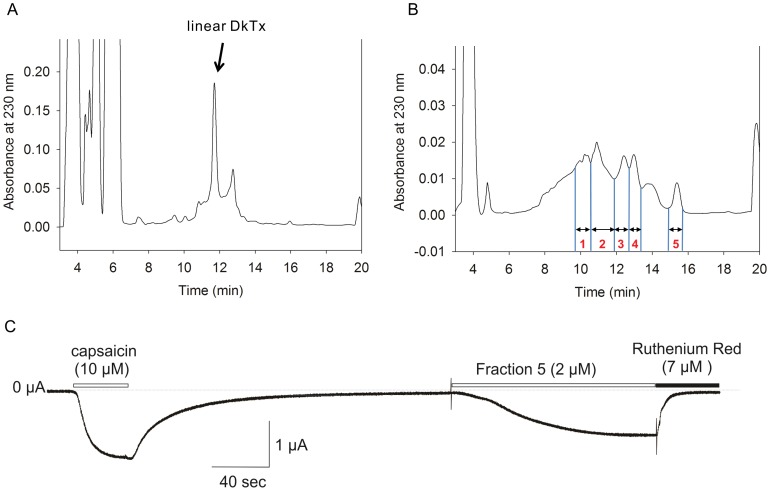
Purification and activity of the DkTx folding product. (A) Purification of linear DkTx. Cleavage of the fusion protein and reduction of the disulfide bond were accomplished using hydroxylamine and DTT, respectively, as described in methods. (B) Linear DkTx was folded in 1 M NH_4_OAc (pH 8.0) buffer containing 1 M GdnHCl, 1 mM EDTA, 2.5 mM GSH and 0.25 mM GSSG for 5 days at 4°C. The toxins were purified using a linear gradient of 29–44% solvent B for 15 min at a flow rate of 14 ml/min, where solvent A was water containing 0.1% TFA and solvent B was acetonitrile containing 0.1% TFA. (C) Fraction 5 activated TRPV1 expressed in oocytes. Holding voltage was −60 mV.

### CD Measurement and Analysis

The circular dichroism (CD) spectra of DkTx, K1 and K2 were measured on a JASCO J–750 spectropolarimeter in 10 mM sodium phosphate (pH 7.0) at 20°C in a quartz cell with a 1-mm path length. The spectra were expressed as molecular ellipticity [θ] in deg •cm^2^ •dmol-1. The secondary structure of DkTx was predicted using the method of Raussens et al. [22].

**Figure 4:**
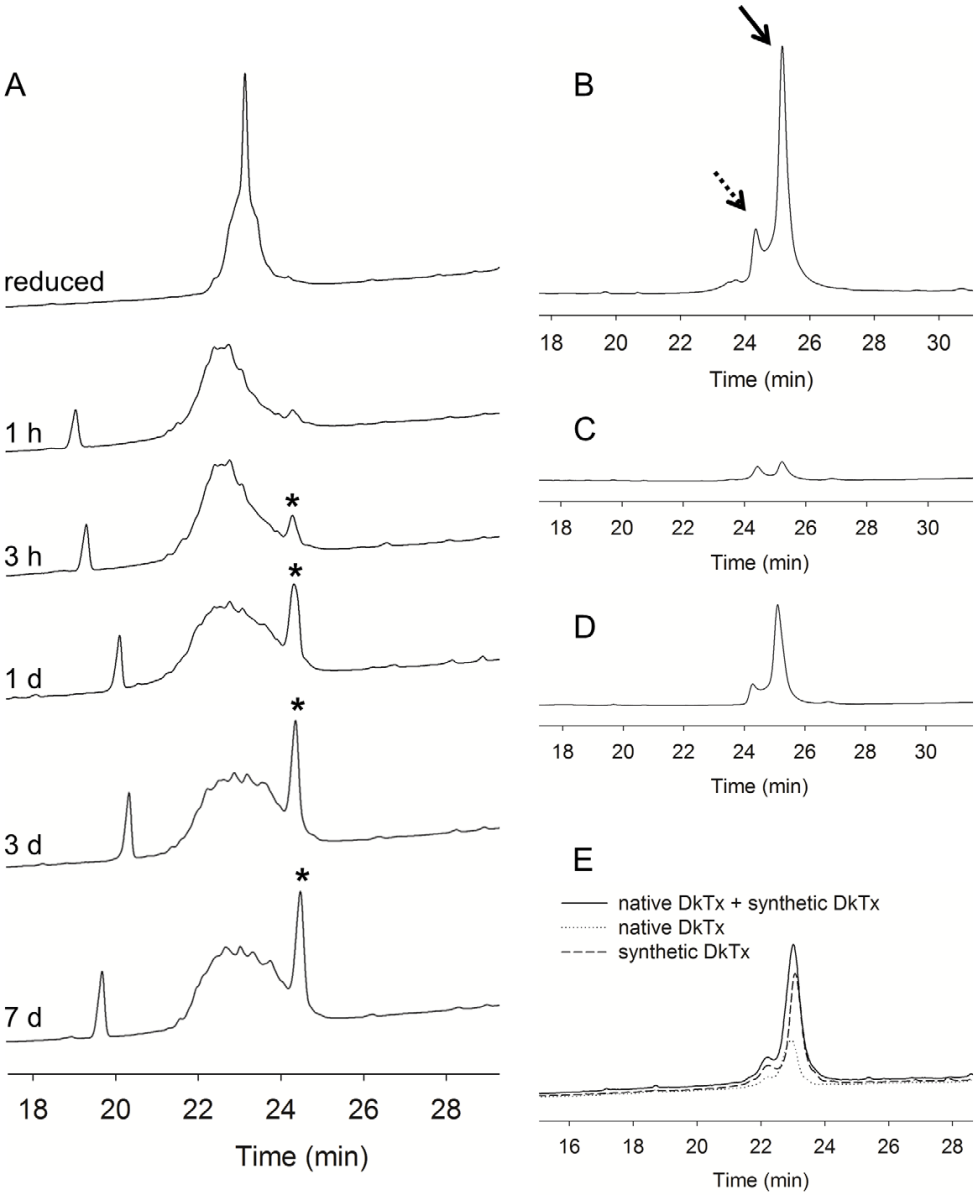
HPLC profiles of DkTx during the folding reaction. The peptides were separated using a linear gradient of 5–65% solvent B for 30 min at a flow rate of 1 ml/min, where solvent A was water containing 0.1% TFA and solvent B was acetonitrile containing 0.1% TFA. (A) Oxidative folding of DkTx in redox buffer was monitored by HPLC. Asterisks indicate correctly folded DkTx. (B) HPLC chromatogram of purified DkTx. Dashed and solid arrows indicate the minor and major forms of DkTx, respectively. (C) The minor form was collected and re-injected after 75 min. (D) The major form was collected and re-injected after 175 min. (E) Co-injection of native and synthetic DkTx.

## Results and Discussion

### Expression of DkTx

To obtain DkTx in the amounts necessary for structural and functional analysis, we expressed the recombinant toxin as a fusion protein in *E. coli*. The artificial gene used for DkTx expression is shown in Figure 1. His-tag, ketosteroid isomerase (KSI) and thioredoxin (TrxA) were each employed as the fusion partner in an effort to identify the best expression conditions. DkTx was produced in an insoluble form when expressed with His-tag or KSI, and the highest expression of DkTx was observed with KSI (Figure 2). Interestingly, when TrxA was used as the fusion partner, DkTx was expressed in a soluble form; however, after cleaving the fusion protein, we found that the expressed soluble DkTx was a misfolded disulfide isomer that differed from the native form and demonstrated no TRPV1 activation (not shown).

**Figure 5:**
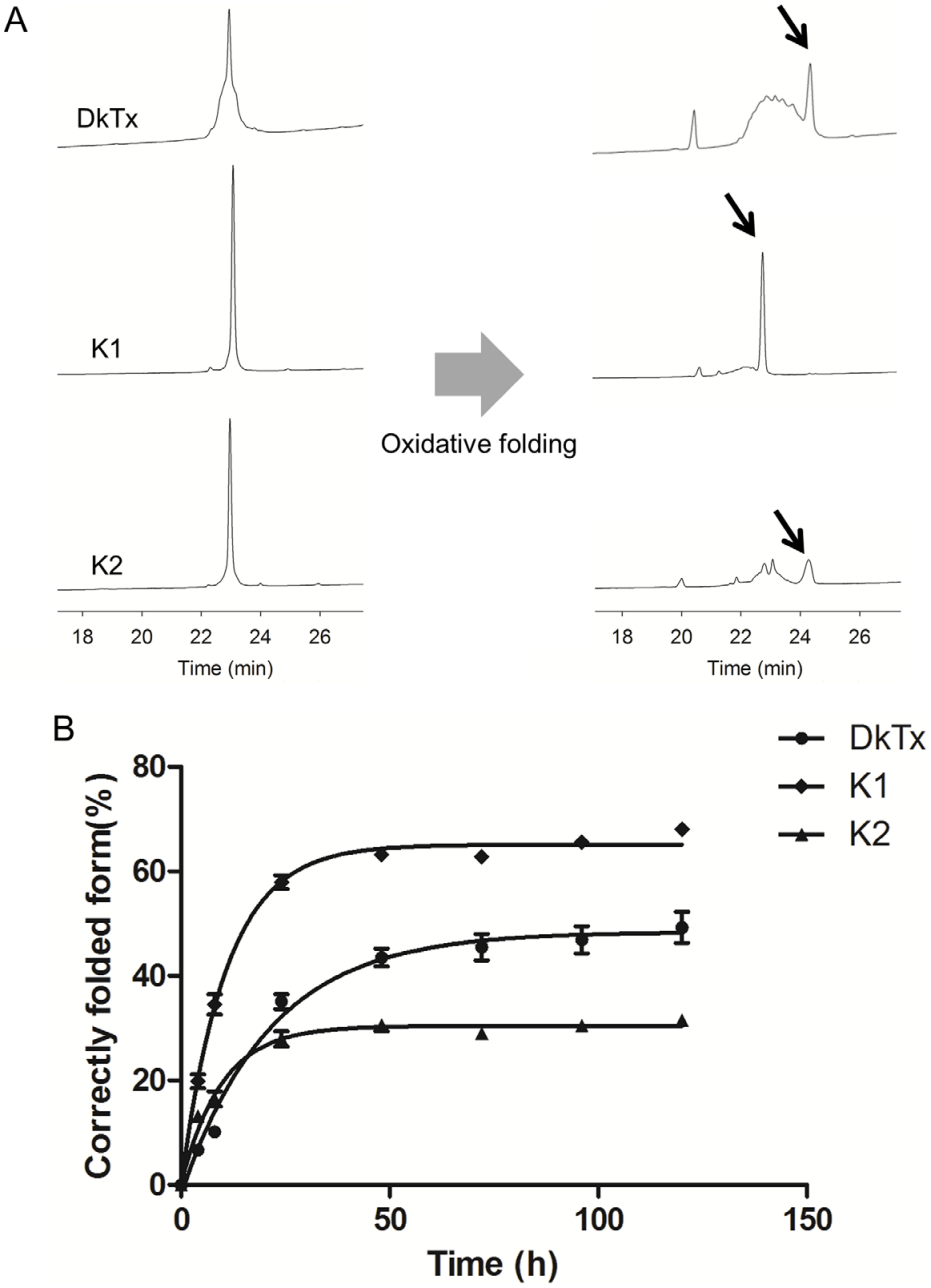
Folding kinetics of K1, K2 and DkTx. (A) HPLC chromatograms showing the folding of DkTx, K1 and K2. The left panel depicts the linear toxins. Arrows indicate correctly folded forms of the toxins. The toxins were eluted with a linear gradient of 5–65% solvent B for 30 min at a flow rate of 1 ml/min, where solvent A was water containing 0.1% TFA and solvent B was acetonitrile containing 0.1% TFA. (B) Comparison of folding kinetics of DkTx, K1 and K2. At each time point, aliquots were withdrawn and acidified by adding acetic acid. Quantification of the folding specifies was accomplished using HPLC. The correctly folded form (%) was determined based on the areas of the HPLC peaks. The curves represent fits of the data to a one-phase association.

Cysteine-rich peptides such as DkTx (which contains 12 cysteines) are usually produced as inclusion bodies in *E. coli* due to the complex disulfide bond connectivity and the reducing environment of the cytoplasm [23], [24]. Although various fusion partners, including TrxA and DsbC, have been employed to express functional forms of cysteine-rich peptides [19], [25], [26], their application to the functional expression of ICK toxins, which contain complex disulfide bond parings, has been rare. Consistent with this pattern, we found that DkTx was produced in a misfolded form in our *E. coli*-based fusion protein expression system. In contrast, however, the use of KSI as the fusion partner resulted in very high yields of linear DkTx, which was successfully oxidatively refolded in redox buffer. In our hands, a typically yield of linear toxin was around 0.4–0.8 mg per L of bacterial culture.

**Figure 6:**
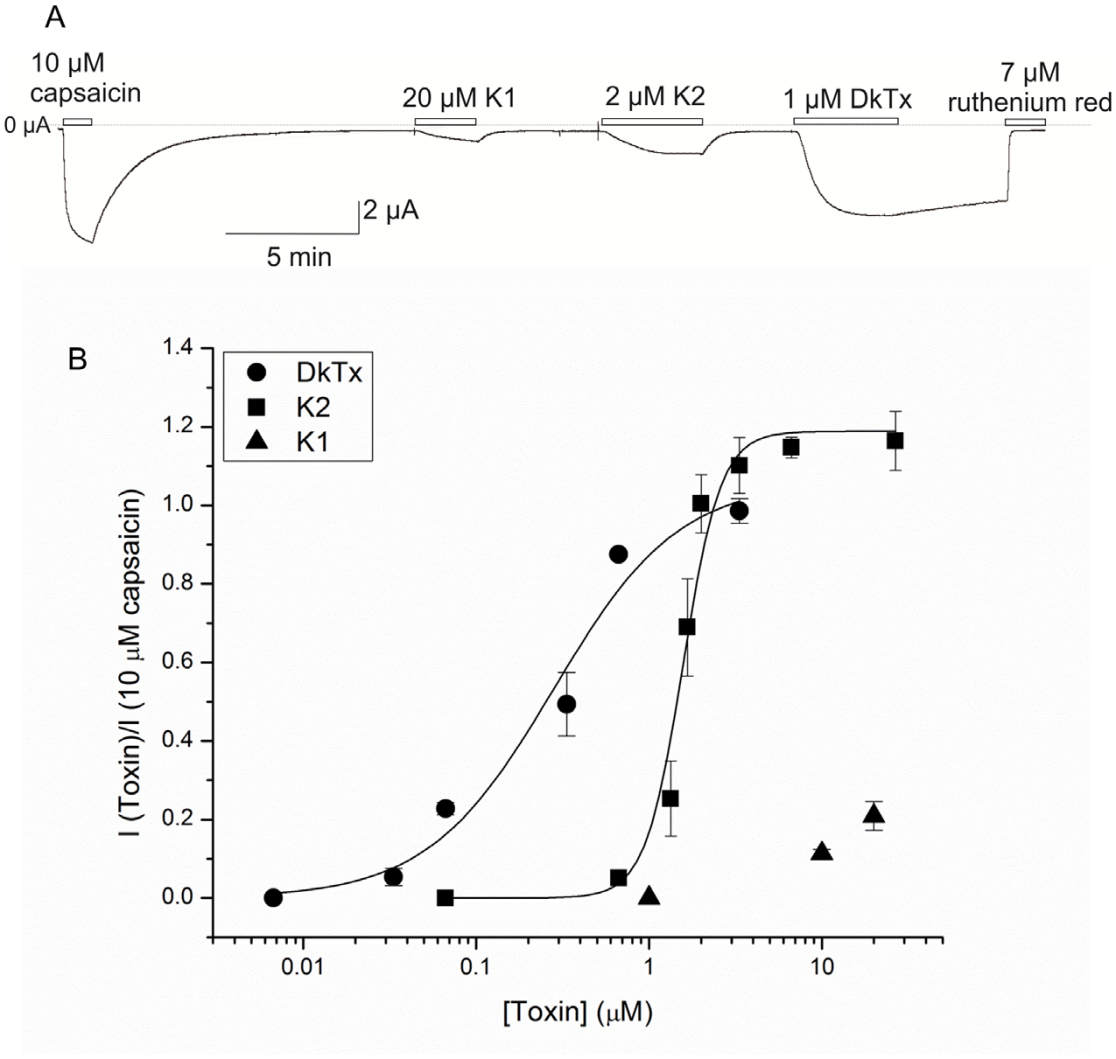
Activity of K1, K2 and DkTx on TRPV1 channels. (A) Synthetic K1, K2 and DkTx activate TRPV1 channels. Oocytes were held at −60 mV and toxins were added to the recording chamber. (B) Concentration-dependence for activation of TRPV1 by the toxins.

### Screening Folding Conditions

We initially refolded linear DkTx in a typical folding solution containing a redox buffer system that is used often for oxidative folding of cysteine-rich peptides (e.g., conotoxin) [27], [28]. As shown in Figure 3B, several isomers were produced upon folding the linear DkTx. To identify the active form(s), the folded product was fractionated using RP-HPLC, and the ability of the individual peaks to activate TRPV1 channels was tested. Upon studying the activity of rTRPV1 expressed heterologously in oocytes we found that fraction 5 robustly activated TRPV1 channels (Fig. 3C). We therefore assumed that this fraction contained the correctly folded form of the toxin.

**Figure 7:**
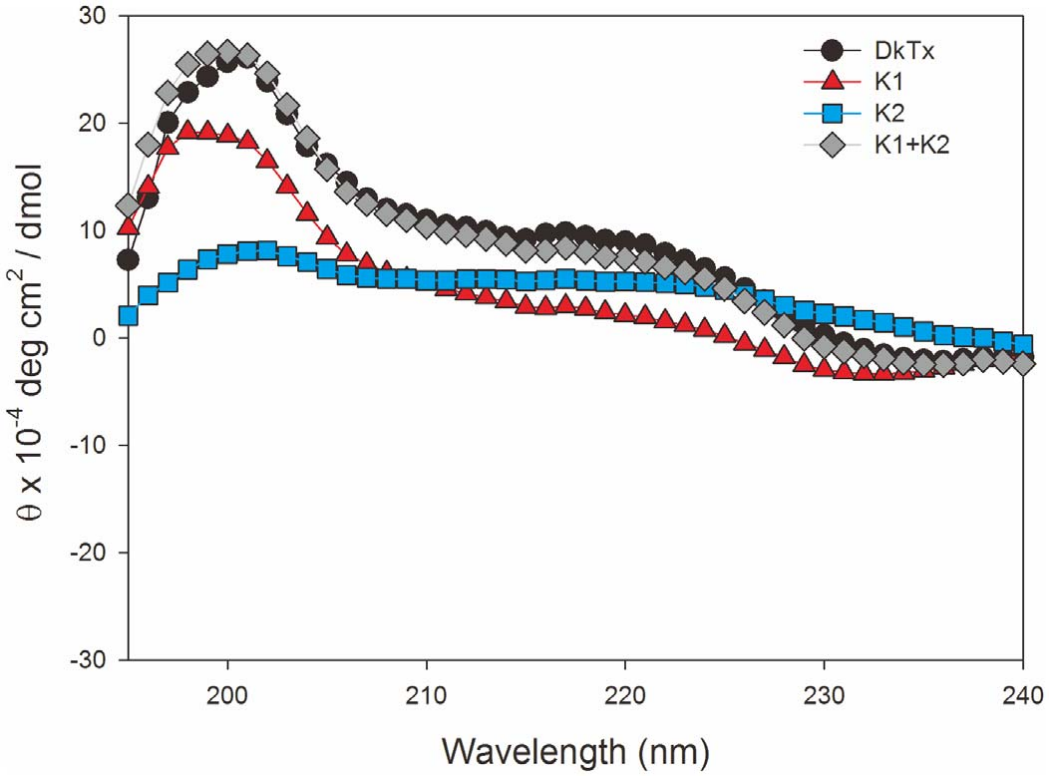
CD spectra of K1, K2 and DkTx. The CD spectra were recorded in 0.01 M sodium phosphate (pH 7.0) at 20°C. K1+K2 depicts the added values of the CD spectra from K1 and K2.

Previous work on the folding of cysteine-rich peptides has revealed that optimizing the folding conditions of these peptides is empirical and differs considerably for each peptide [29], [30]. Upon attempting refolding in the typical folding solution used for refolding conotoxin, we obtained poor yields (∼2% correctly folded). Therefore, we screened various folding conditions by changing the buffer and temperature and by introducing several folding additives such as guanidine, glycerol, and CH_3_CN in an effort to improve the folding yield. We also tested the effect of increasing the hydrophobicity of the solution using organic solvents or glycerol on the refolding yield since we observed that correctly folded DkTx is more hydrophobic than the linear form (Figs. 3, 4). The folding conditions tested and the results obtained are summarized in Table 1. Notably, a significant increase (up to 10-fold) in yield was observed when detergent was added to the folding solution. Although, the best folding yield was obtained with 0.5% Fos-choline (∼30%), we decided to use Triton X-100 (which resulted in ∼24% yield) for large sample preparation due to the prohibitively high cost of Fos-choline. Low temperature and low peptide concentration were also important factors that contributed to efficient DkTx folding.

Detergents have been previously reportedly to be useful additives for the folding of cysteine-rich peptides such as delta-conotoxin TxVIA, SVIE and GmVIA [31]. Interestingly, these peptides have large hydrophobic surface areas, and the native form is more hydrophobic than the linear or disulfide bonded isomers–a characteristic that we also observed in DkTx. The omega-conotoxin TxVII, which carries a net negative charge and is very hydrophobic, is a good example in which folding yield is closely related to the protein’s hydrophobicity [32]. Correctly folded TxVII is more hydrophobic than the reduced form, and the folding yield of the toxin is dramatically increased in the presence of hydrophobic solvents such as CH_3_CN or MeOH. Moreover, it has been suggested that the hydrophobic residues of the toxin are important for its channel binding [33]. Based on these findings, it seems likely that the correctly folded form of DkTx contains a large hydrophobic surface area, which could be related to the function of the toxin. It appears that the surface exposure of hydrophobic amino acids is critical for the correct folding of DkTx, and the presence of detergents render such exposure thermodynamically favorable.

### Optimal Oxidative Folding of DkTx

Linear DkTx was folded for 5 days in a folding solution containing 0.4 M Tris-HCl, 0.5% Triton X-100, 2.5 mM GSH, 0.25 mM GSSG and 1 mM EDTA. The product was then purified using SP-5PW cation exchange column followed by RP-HPLC. When the purified synthetic DkTx was co-injected for HPLC with native DkTx from the venom of *O. huwena*, the toxins co-eluted (Fig. 4E). In addition, the synthetic DkTx exhibited an intriguing chromatographic phenomenon: although the ratio between the minor and major peaks varied from 1∶1 to 1∶6 (Figure 4B–E), it always eluted in two peaks, which is reminiscent of hanatoxin1 (HaTx1) [10]. When the minor peak was collected and re-injected after 75 min, the ratio between the minor and major forms was 1∶1 (Fig. 4C). Reinjection of the major form also yielded both the major and the minor form (Fig. 4D). These results suggest that DkTx exists in an interconvertible conformation, and the major conformation is more favorable in aqueous solution. Although we tried several different temperatures and pHs (data not shown), we were unable to identify a set of conditions under which the minor conformation predominated over the major one a equilibrium.

### Synthesis of K1 and K2

To better understand the mechanism of interaction between DkTx and TRPV1, we chemically synthesized the two knot domains (K1 and K2) of DkTx separately. As shown in Figure 5A, a single peak containing the folding product was observed after oxidative folding of K1. In contrast, with K2 a correctly folded form was observed along with other disulfide bond isomers. This folding pattern of K2 was similar to that of DkTx, in which the correctly folded form was more hydrophobic than the linear or misfolded forms. In addition, although K1 and K2 share high sequence homology (67% identity), their folding characteristics were quite different. First, the folding yield of K1 (∼65%) was about 2 times higher than that of K2 (∼30%). Second, whereas K1 efficiently folded in redox buffer, without detergent or organic solvent, folding K2 under hydrophilic conditions without detergent resulted to the formation of a large precipitate and a variety of disulfide bond isomers, thereby decreasing the folding yield (not shown). It was previously reported that although SGTx1 and HaTx1, two well-studied gating modifiers of the Kv2.1 channel, share high primary sequence homology (78% identity), the folding yield of HaTx1 (<1%) is very much lower than that of SGTx1 (∼60%) [6]. In addition, substitution of alanine for various residues in SGTx1 showed that its folding could be significantly influenced by mutation of single amino acids, which then prevented identification and isolation of the correctly folded toxin [34]. It is thus plausible that subtle differences in a primary sequence can dramatically alter folding properties. Therefore, despite the high degree of sequence homology shared by K1 and K2, the small difference in their primary sequences could substantially affect their folding yields.

### Folding Kinetics

We were intrigued by the observation that the folding yield of K2 was lower than the yield of DkTx, suggesting that K1 influences the folding of K2 in the full-length toxin To investigate the folding of K1, K2 and DkTx in more detail, we measured the folding kinetics of the toxins based on the areas of their HPLC peaks at different time points during the folding reactions. These kinetic studies were carried out in the same optimal folding solutions employed in the synthesis of DkTx. We found that the data were well fitted by single exponential functions which were then used to obtain rate constants for folding (Figure 5B; Table 2) The rate constants for DkTx, K1 and K2 were 0.046 h^−1^, 0.092 h^−1^ and 0.103 h^−1^, respectively, and the steady-state accumulations of the folded forms were 50.20%, 65.10% and 30.16%, respectively. It is noteworthy that the folding yield of DkTx was higher than that of K2, a result that would not be expected if K1 and K2 fold independently, because in that case the folding yield of DkTx should be governed by that of K2. However, comparison of the yields and rate constants of DkTx and K2 suggests that K2 undergoes a different folding mechanism in the presence of K1 and it appears that K1 helps the folding of K2. We also performed experiments in which linear K1 and K2 were mixed and folded together, and we observed that the folding kinetics of K2 did not change (not shown). Based on our findings that K1 is more soluble and less active toward the TRPV1 channel than K2, we suggest that two functions of K1 within DkTx would be to act as a molecular chaperone to aid folding of K2, and to increase the water solubility of the toxin.

### Voltage Clamp Recording of the Effects of K1, K2 and DkTx on TRPV1 Activity

The functional activities of DkTx, K1 and K2 were also compared by recording their electrophysiological effects on TRPV1-expressing cells. Application of 1 µM DkTx evoked large currents that decayed slowly following removal of the toxin from the recording chamber and were blocked by ruthenium red (Figure 6A). In contrast to what was observed with DkTx, activation of TRPV1 by K1 and K2 was readily reversible (Fig. 6A). We also examined the concentration-dependence for toxin activation of TRPV1 and observed that DkTx was the most potent TRPV1 agonist among the three toxins examined, and that K2 was more active than K1 (Figure 6B). All of these results are consistent with what was previously reported for full-length DkTx and for K1 and K2 prepared by enzymatic cleavage between the two knots [17]. The EC_50_ for DkTx and K2 was found to be 0.28 µM and 1.57 µM, respectively. Interestingly, the Hill coefficients obtained for DkTx-activation of TRPV1 was near 1, whereas that for K2 was about 4, consistent with previous calcium imaging experiments [17]. These results demonstrate that K2 activation of TRPV1 is highly cooperative, implying that several K2 molecules bind to one molecule of TRPV1 to orchestrate channel activation. When K1 and K2 were applied to the A657P mutant of TRPV1, which was reported to be insensitive to DkTx [17], neither toxin activated the channel (data not shown). Taken together, these results indicate that K1, K2 and DkTx bind to equivalent sites on the receptor and that bivalency is important for the efficient and slowly reversible activation by DkTx.

### Circular Dichroism

We next used CD spectroscopy to analyze the secondary structure of DkTx. As shown in Figure 7, the CD spectrum of DkTx has a maximum near 201 nm and a minimum near 235 nm, similar to what has been observed with related ICK tarantula toxins (ref 34). Analysis with a secondary structure estimation program revealed the α-helix, β­­-sheet and β-turn contents of DkTx to be 20.6%, 38.5% and 12.4%, respectively. Interestingly, adding the CD spectra of K1 and K2 gave a pattern similar to the CD spectrum of DkTx, suggesting the secondary structures of K1 and K2 are well conserved, even when they are separated into separated knots.

In an earlier paper (17), K1 and K2 were obtained by cleaving DkTx-HYR, a modified DkTx containing a Genenase I cleavage site (His-Tyr-Arg) in the linker region. By contrast, in the present study, K1 and K2 were chemically synthesized separately. Nonetheless, the activities of the two toxins were similar to those previously reported, suggesting K1 and K2 can each be separately synthesized in their functional form, without the help of the other knot. On the other hand, the double peak phenomenon of DkTx, which is reminiscent of HaTx1 (43), was completely lost when it was cleaved into separate knots. It has been suggested that the conformational heterogeneity of HaTx1 reflects the rotational isomerization of a disulfide bond, and this may also be the case with DkTx. Alternatively, the isomerization of DkTx may be produced via cis and trans isomerization of the short linker, which contains two Pro residues. To investigate this conformation heterogeneity, a NMR study of the three-dimensional structures of K1, K2 and DkTx is currently in progress.

In summary, we have synthesized DkTx, optimized its folding conditions, and tested the activity of the folded protein on TRPV1 channels. We also synthesized K1 and K2, and our studies on the folding of K1, K2, and DkTx suggests that the two domains do not fold independently; rather, they influence the rates of folding of each other during the folding process. The CD spectra of K1 and K2 showed that the overall secondary structure of DkTx is well maintained, even when it is cleaved into separated knots. These studies should contribute to improving the synthesis of DkTx and thus provide a valuable tool for the study of TRPV1 channels as well as further structural and functional analyses of DkTx and TRPV1.
